# Supplementary material for: Deep transcriptome annotation enables the discovery and functional characterization of cryptic small proteins
Source: eLife. 2017 Oct 30;6:e27860. doi: 10.7554/eLife.27860 (PMC5703645; doi:10.7554/eLife.27860)
Supplement: Transparent reporting form [file elife-27860-transrepform.pdf]

1st Floor
24 Hills Road
Cambridge CB2 1JP, UK

P 01223 855340
W elifesciences.org
T @elife

eLife's transparent reporting form

We encourage authors to provide detailed information *within their submission* to facilitate the interpretation and replication of experiments. If you have any questions, please contact us: editorial@elifesciences.org.

Sample-size estimation

- You should state whether an appropriate sample size was computed when the study was being designed
- You should state the statistical method of sample size computation and any required assumptions
- If no explicit power analysis was used, you should describe how you decided what sample (replicate) size (number) to use

Please outline where this information can be found within the submission (e.g., page numbers or figure legends), or explain why this information doesn't apply to your submission:

This information can be found here:
Figure 1f: legend.
Figure 3: Results page 7 and Materials and methods page 23
Figure 11: Materials and methods page 21, and Table 4
Figure 12 and Figure 12-figure supplement 2: Results page 11 and Materials and methods page 27 and Legends.
Figure 13, Figure 13-figure supplement 3, Figure 13-figure supplement 4: Legend.
Figure 14: Legend.
Figure 15: Legend and Results page 15.
Figure 1-figure supplement 2: Legend

Replicates

- You should report how often each experiment was performed
- You should include a definition of biological versus technical replication
- The data obtained should be provided and sufficient information should be provided to indicate the number of independent biological and/or technical replicates
- If you encountered any outliers, you should describe how these were handled
- Criteria for exclusion/inclusion of data should be clearly stated
- High-throughput sequence data should be uploaded before submission, with a private link for reviewers provided (these are available from both GEO and ArrayExpress)

eLife Sciences Publications, Ltd is a limited liability non-profit non-stock corporation incorporated in the State of Delaware, USA, with company number 5030732, and is registered in the UK with company number FC030576 and branch number BR015634 at the address 1st Floor, 24 Hills Road, Cambridge CB2 1JP | August 2014

Please outline where this information can be found within the submission (e.g., page numbers or figure legends), or explain why this information doesn't apply to your submission:

Figure 1f: the hg38 transcriptome was shuffled 100 times, indicated in the legend.
Figure 13: n=3, indicated in the legend.
Figure 14: n=3, indicated in the legend.
Figure 15: n=2 (10 cells each), indicated in the legend

Statistical reporting

- Statistical analysis methods should be described and justified
- Raw data should be presented in figures whenever informative to do so (typically when N per group is less than 10)
- For each experiment, you should identify the statistical tests used, exact values of N, definitions of center, methods of multiple test correction, and dispersion and precision measures (e.g., mean, median, SD, SEM, confidence intervals; and, for the major substantive results, a measure of effect size (e.g., Pearson's r, Cohen's d)
- Report exact p-values wherever possible alongside the summary statistics and 95% confidence intervals. These should be reported for all key questions and not only when the p-value is less than 0.05.

Please outline where this information can be found within the submission (e.g., page numbers or figure legends), or explain why this information doesn't apply to your submission:

This information can be found here:
Figure 1f: legend.
Figure 3: Results page 7 and Materials and methods page 23
Figure 11: Materials and methods page 21, and Table 4
Figure 12 and Figure 12-figure supplement 2: Results page 11 and Materials and methods page 27 and Legends.
Figure 13, Figure 13-figure supplement 3, Figure 13-figure supplement 4: Legend.
Figure 14: Legend.
Figure 15: Legend and Results page 15.
Figure 1-figure supplement 2: Legend

(For large datasets, or papers with a very large number of statistical tests, you may upload a single table file with tests, Ns, etc., with reference to page numbers in the manuscript.)

Additional data files ("source data")

- We encourage you to upload relevant additional data files, such as numerical data that are represented as a graph in a figure, or as a summary table

- Where provided, these should be in the most useful format, and they can be uploaded as "Source data" files linked to a main figure or table
- Include model definition files including the full list of parameters used
- Include code used for data analysis (e.g., R, MatLab)
- Avoid stating that data files are "available upon request"

Please indicate the figures or tables for which source data files have been provided:

Figures 1-12, Figure 1-figure supplement 1, Figure 1-figure supplement 2, Figure 8-figure supplement 1,

Homo sapiens alternative protein predictions based on RefSeq GRCh38 (hg38) based on assembly GCF_000001405.26: https://roucoulab.com/p/downloads

Pan troglodytes alternative protein predictions based on annotation version 2.4.1: https://roucoulab.com/p/downloads

Mus musculus alternative protein predictions based on annotation version GRCm38: https://roucoulab.com/p/downloads

Bos taurus alternative protein predictions based on annotation version 4.6.1: https://roucoulab.com/p/downloads

Xenopus tropicalis alternative protein predictions based on Ensembl annotation JGI_4.2: https://roucoulab.com/p/downloads

Danio rerio alternative protein predictions based on Ensembl annotation Zv9.79: https://roucoulab.com/p/downloads

Drosophila melanogaster alternative protein predictions based on annotation version 6.07: https://roucoulab.com/p/downloads

Caenorhabditis elegans alternative protein predictions based on annotation version WS251: https://roucoulab.com/p/downloads

Saccharomyces cerevisiae S288c alternative protein predictions based on RefSeq annotation R64 assembly GCF_000146045.2: https://roucoulab.com/p/downloads
